# “It was all yellow” first patient with resistant depresion treated with esketamina

**DOI:** 10.1192/j.eurpsy.2024.1100

**Published:** 2024-08-27

**Authors:** M. J. Mateos - Sexmero, B. Arribas - Simón, T. Jiménez - Aparicio, Ó. Martín - Santiago

**Affiliations:** Psychiatry, Hospital Clínico Universitairo de Valladolid, Valladolid, Spain

## Abstract

**Introduction:**

Esketamine, an active Ketamine isomeric form that indirectly inhibits the GABAergic neuronal pathways, has been recently approved to treated severe, resistant depressive disorders. Here, we present the case of a 64 years old woman diagnosed with severe, resistant depression and an initial score of 28 points in the Hamilton Depression Rating Scale who was treated with Esketamine with excellent response and a HDRS of 8 points after 4 months.

**Objectives:**

To expose our experience with the first patient treated with Esketamine in our Hospital.

**Methods:**

Describing the patient’s patobiography and the different treatments lines tried in first place and exposing the experience among Ketamine treatment and the final results.

**Results:**

We present the case of a 64 years old woman, divorced and retired, who lives with her son since the aggravation of the depressive symptomatology, with no medical nor surgical background and no history in Mental Health before her first psychiatric internment in 2020. Between February 2020 and June 2023, 5 different treatments options with supervise intake were tried, including increment of the dose, antidepressant rotation, the combination of Desvenlafaxine + Mirtazapine and adding Topiramate and Lithium, with no improvement. Among this years, 3 psychiatric internments were needed because of the depressive symptoms and 1 more hospitalization in Internal Medicine was required because of the patients severe, malnutritional state. In June 2023 and after two complete analysis, a MR and a score of 28 points in the Hamilton Depression Rating Scale treatment with Esketamine was started with no incidences. She described one dissociative episode during which she assures “she was surrounded by soft, rubbery, yellow bubbles”. After 4 months of treatment the patient has recovered her previous functional rate and has an 8 points score in the HDRS.

**Conclusions:**

In conclusion, we can affirm that Esketamine is an effective and secure option for Resistant Depresion Dissorder. Nevertheless, Before considering a Depressive Episode as “resistant to treatment”, treatment adherence and other medical, surgical and psychiatric comorbidities must be studied.

**Disclosure of Interest:**

None Declared

